# Egg Yolk Immunoglobulins (IgY) Purification, Activity Enhancement, and Potential Benefits for Human Health

**DOI:** 10.3390/nu17172890

**Published:** 2025-09-06

**Authors:** Huilong Qiu, Xiaomin Jin, Xiaomei Zhang, Ke Chen, Lianshun Wang, Jiaqiang Huang

**Affiliations:** 1 Department of Nutrition and Health, China Agricultural University, Beijing 100083, China; qiucau2580@163.com (H.Q.); b20243050504@cau.edu.cn (K.C.); 2 College of Marine Resources & Environment, Hebei Normal University of Science & Technology, Qinhuangdao 066600, China; xicom@163.com; 3 Zaozhuang Animal Husbandry and Fishery Development Center, Zaozhuang 277800, China; 15665218608@163.com; 4 School of Fisheries and Life Science, Dalian Ocean University, Dalian 116023, China; wanglianshun@dlou.edu.cn

**Keywords:** egg yolk immunoglobulins (IgY), purification, activity enhancement, health benefits

## Abstract

With the rapid development of the laying hen industry and the continuous innovation of farming technology, egg production continues to increase. Eggs are rich in proteins, lipids, and immunoglobulins (IgY) found in the egg yolk. IgY can be used to treat human diseases and prevent bacterial and viral infections. In addition, IgY has advantages over IgG in biological structure and function and is characterized by high specificity, safety, yield, and economic efficiency. This review describes the basic structure and properties of IgY, lists a variety of IgY purification methods, and outlines measures to maintain and enhance its activity, focusing on the current status of its research in immunoprevention and treatment of human diseases and outlines its importance, and finally proposes the current challenges and future research priorities of IgY in the field of biomedical research to provide a scientific basis for the wide range of applications of IgY in human health.

## 1. Introduction

**Figure 1:**
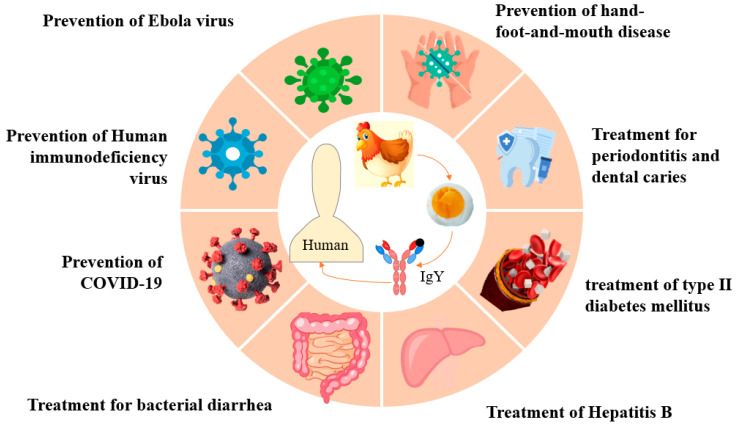
Potential applications of IgY in human health.

The chicken belongs to the order *Galliformes* and is one of the earliest animals to be domesticated by humans. It is also one of the most important sources of protein for humans. In recent years, the laying hen industry has undergone 20 years of rapid development, and global egg production has continued to increase. As consumers emphasize a healthy diet, the demand for high-quality, pollution-free eggs grows, and researchers are conducting extensive studies on the various protein fractions in eggs to deeply explore their potential application value [1]. In 1960, scientists found that a large number of antibodies similar to mammalian immunoglobulin (IgG) existed in the egg yolk of laying hens after antigen immunization. This antibody is chicken egg yolk immunoglobulin (IgY), which is generated by a series of immune responses after the stimulation of hens with foreign antigens. The IgY antibodies pass through the bloodstream into the egg yolk and are stored in the egg yolk. It is an immunoglobulin that primarily exists in the yolk and is characterized by high specificity, high safety, easy accessibility, and low acquisition cost [2]. IgY and IgG differ in terms of constant structural domains, heavy chains, side chains, and molecular size, which results in variations in activity and several biological activities. Under laboratory conditions, studies have shown that IgY has potential benefits in the treatment of human diabetes mellitus, human immunodeficiency virus (HIV), ovarian cancer, dental caries, viral hepatitis type B (HBV), diarrhea, hand-foot-and-mouth disease (HFMD), and ebola virus (EBOV), as well as showing varying degrees of immunoprophylaxis against enteroviruses and COVID-19 (Figure 1). In addition, in animal health, it is beneficial in preventing pathogenic bacterial and viral infections, regulating intestinal flora to alleviate diarrhea and improving growth performance [3]. Domestic and foreign scholars continue to explore the medical value of IgY; it is expected that it will be widely used in the diagnosis and prevention of human and livestock diseases in the future. Scientific and reasonable protection of IgY, dosage form design, and delivery are crucial to transform functional IgY antibodies into desired IgY products for therapeutic and prophylactic administration [4].

In summary, we describe the structural features of IgY and its functional differences with IgG, summarize the various methods of IgY extraction and measures to enhance its biological activity, review the progress of its application in the prevention and treatment of a variety of human diseases, and present the current challenges faced by IgY and future research directions. The purpose of this paper is to provide scientific evidence for the broad application of IgY in the prevention and treatment of human diseases, thereby offering subsequent scientists clear research directions.

## 2. Structure and Functions of IgY

### 2.1. Biological Structure

IgY consists of two heavy chains (H chains) and two light chains (L chains) connected by disulfide bonds to form a stable Y-shaped tetrapeptide structure. The CH region of IgY contains glycosylation sites, and glycosylation plays an important role in the stability and solubility of the antibody. The *N*-glycosylation chain of IgY contains two glyco-structures, namely, the high-mannose chain and the composite glycan chain, and it is predominantly a high mannose chain, with the presence of sialic acid and galactose modifications at the end of the glycan chain. In addition, IgY has two functional regions, Fab and Fc, with a short region rich in proline and glycine residues between the Fab and Fc segments [5]. Rather than acting independently, the Fab and Fc regions achieve efficient immune defense through a synergistic mechanism of specific binding-effect mediation. Compared to IgG, IgY has a longer heavy chain and a higher molecular weight of 180 kDa compared to 150 kDa for IgG, and IgY has more glycosylated side chains and constant structural domains, which are more hydrophobic and inhibit hydrolysis of the protein catalyzed by proteinase. In addition, IgY has no hinge region between CH1 and CH2, which cannot undergo conformational changes, is less flexible, and has additional CH4 structural domains.

### 2.2. Biochemical Functions

IgY is similar to other antibodies in that the variable region possesses the ability to bind specifically to antigens and the Fc region has an immunomodulatory function. The Fc binds specifically to three characterized Fc receptors, such as chicken Ig-like receptor AB1 (CHIR-AB1), the chicken yolk sac IgY receptor (FcRY), and Gallus gallus Fc receptor (ggFcR) [6,7]. Its Fc region cannot interact with mammalian Fc receptors (IgG-FcγR and IgE-FcεR), rheumatoid factor (RF), complement factor (CF), Staphylococcal protein A, Streptococcal protein G, and Peptostreptococcal protein L [8]. Based on the fact that IgY cannot bind to mammalian immunomodulatory proteins, it does not interact in immune recognition and disease treatment, reduces endogenous interference and cross-reactivity effects, and is valuable in immunotherapy and prophylaxis.

## 3. Characteristics and Action Mechanisms of IgY

### 3.1. Biological Characteristics

IgY has strong heat, alkaline, and enzyme degradation resistance and immunological properties. It still holds activity in an acidic environment, can be heated to 60 °C in the presence of protein hydrolyzing enzymes, and has excellent thermal stability. IgY remained stable below 70 °C but lost significant activity when the temperature exceeded 70 °C. IgY was relatively stable within the pH range of 4 to 10, and it had a certain degree of resistance to pepsin and trypsin [9]. IgY is more resistant to pepsin but more sensitive to trypsin, and the activity of IgY remained at 39% and 41% after 8 h of a mixed reaction with trypsin and pancreatic rennet protease, respectively [10]. Additionally, IgY is more stable than IgG at 60 °C. This is because IgY’s heavy chain contains the CH4 structural domain, which is crucial for molecular stability [11]. Glycosylation is one of the very important post-translational modifications of proteins, immunoglobulins belong to glycoproteins, and IgY mainly relies on *N*-glycosylation to resist hydrolysis by pepsin and papain [12]. IgY has more glycosylation sites than IgG molecules and is distributed in different regions of IgY. Glycosylation modifications enhance the structural stability of IgY, and the presence of *N*-glycosylation chains can place the IgY resistance to guanidine hydrochloride-induced defolding by 0.6 M, which is about 0.1 M guanidine hydrochloride higher than that of IgG. Glycosylation modifications also prolong the half-life of immunoglobulins in serum [13]. It has a stronger immune response to antigens in mammals and is specific for antigen detection, so laying hens can be used as a source of animals for the production of high-quality antibodies.

### 3.2. Immune Mechanisms

Chickens have both cellular and humoral immune systems that are regulated by the thymus and the bursa, respectively. When stimulated by foreign antigens, B cells differentiate into plasma cells that secrete specific antibodies which bind to the antigen to produce immunoglobulin. These antibodies gradually accumulate in the oocytes of chickens to form IgY. With the help of oocyte membrane receptors, IgY is then transported from the maternal serum to the egg yolk. IgY enters the bloodstream of the chicken embryo during egg incubation and provides a specific immune protective barrier against disease in the chick. Immunoglobulins are mainly used to remove pathogenic microorganisms from the organism by binding to antigens, thus achieving the purpose of the organism’s defense against bacterial and viral aggression. IgY promotes specific immune responses during organismal infections mainly through two pathways [14,15]. The first one can directly adhere to its cell wall, destroying the integrity of pathogenic microorganisms and directly inhibiting the reproduction of pathogenic bacteria. The second method is to adhere to the bacterial hairs. This prevents them from attaching to the intestinal mucosal epithelial cells.

## 4. Extraction and Activity Enhancement of IgY

### 4.1. Methods for IgY Isolation and Purification

The main components in egg yolk are proteins and fats. Livetin is an important protein constituent in egg yolk, accounting for about 9.3% of the dry matter of the yolk, and it exists in the yolk in the form of α-, β-, and γ-yolk globulins in three forms. α-livetin’s main component is albumin, the β-livetin’s main component is the α-2-glycoprotein, and the γ-livetin main component is Immunoglobulin Y [16]. Due to the difficulty of removing the lipid fraction of egg yolk as well as the immaturity of the purification process of IgY, chloroform is traditionally used to denature the lipoproteins in egg yolk and then remove them by centrifugation. However, the yield of recovered IgY in this method is low, and the chloroform is highly toxic and can even be carcinogenic if too much is ingested. In recent years, IgY isolation and purification methods have been improved, with convenient operation and higher extraction purity, such as ammonium sulfate precipitation (AMS), polyethylene glycol (PEG), ethanol, ultrafiltration, ion exchange chromatography (IEC), and water dilution (Table 1). It has been shown that the purification of IgY by the PEG precipitation of egg yolk and affinity chromatography based on human mycoplasma proteins with protein M can significantly increase product content [17]. As people continue to explore the biomedical value of IgY, efficient and standardized extraction and purification methods can meet the needs of actual industrial production. IgY polyclonal antibodies play a pivotal role in the field of academic research due to their unique advantages, such as high specificity and abundant yield. These advantages drive the continuous advancement of isolation and purification techniques.

### 4.2. Enhancement of IgY Activity

In recent years, there has been widespread attention on exploring ways to enhance IgY activity. Therefore, selecting appropriate antigens and optimizing the immunization program for chickens is useful for enhancing IgY activity. This includes the immunization dose, frequency, and interval, as well as the antigen emulsification process. Efficient purification methods effectively remove other proteins and impurities from the yolk, increasing antibody purity and indirectly enhancing IgY activity. It has been found that the use of adjuvants to improve immunity in laying hens can increase the yield and activity of antibodies in egg yolk, Freund’s incomplete adjuvant (FIA) and C-phosphate–guanosine oligodeoxynucleotides (CpG-ODN) can enhance the immune response against the interleukin-10 (IL-10) peptide in laying hens and increase the antibody titer in egg yolk [30]. In addition, liposome embedding, microencapsulation, stabilizer development, glycosylation modification, and IgY monoclonal antibody production show potential advantages in enhancing IgY affinity, prolonging the half-life of antibodies, and improving utilization efficiency.

Affinity is a measure of the strength of the interaction between an antigen and an antibody, and its magnitude directly affects the function of the antibody. The higher the affinity, the longer the antigen–antibody interaction and the further the antibody triggers a biological response to clear the antigen [31]. IgY was embedded in liposomes with lecithin and cholesterol and incubated in pepsin solution at pH 3 for 1 h at 37 °C, and 80% of the activity of IgY was still preserved, suggesting that liposome embedding can assist IgY in resisting acidic environments and inhibiting pepsin hydrolysis [32]. The microencapsulation of IgY in egg yolk with 20% β-cyclodextrin and gum arabic can effectively protect the bioactivity of IgY from inactivation due to pepsin hydrolysis [33]. Embedding IgY in chitosan and alginate microcapsules significantly improves its stability in simulated gastric fluid pH 1.2, and the residual antibody activity is unaffected by the pH of the embedding medium [34]; when IgY was encapsulated in alginic acid and carrageenan microgels, it was able to exert normal biological activity in the simulated gastrointestinal tract [35], demonstrating that microencapsulated IgY is resistant to hydrolysis by pepsin. Sorbitol, a polyol commonly used to enhance protein stability [36], was found to maintain the stability of IgY under acidic conditions by enhancing hydrophobic interactions and encapsulating the pepsin protein hydrolysis site, preventing the exposure of its aromatic and carboxylic acid amino acid residues. The acid-induced stability of IgY was significantly enhanced in 30% sorbitol solution when the pH was 3. In 50% sorbitol solution, the activity of IgY was not affected by pH, high concentration of sucrose maintained the stability of IgY, and high activity was retained in 50% sucrose solution at 80 °C [37]. Therefore, sorbitol and sucrose can serve as effective stabilizers of IgY under acidic conditions.

The extraction of specific egg yolk antibodies and application in a chitosan coating containing IgY increases the microbial and sensory quality of fish flesh at 4 °C [38]. However, glycosylation of the conserved asparagine residue in each heavy chain of IgG in the CH2 domain is known as *N*-glycosylation [39]. Glycosylation modification can also enhance the structural stability and biological activity of proteins. The chemical modification of protein by methoxy polyethylene glycol (mPEG) was studied, and the results showed that the modified protein kept its primary structure intact, had better stability in pepsin and trypsin solutions, and better resistance to acid and alkali than the unmodified natural protein [40]. The IgY primary structure was kept intact. The glycosylation modification of IgY using the Melad reaction showed that the thermal denaturation temperature of glucosylated IgY was as high as 79.8 °C, and the immunoreactivity increased by 30.3%, which proved that monoglycosylation modification could improve thermal stability and immunoreactivity in the gastric fluid of IgY [41]. It is one of the most common post-translational modifications and important critical quality attributes of monoclonal antibody therapeutics. The recombinant monoclonal IgY antibodies (mIgY) after hybridoma technology and phage characterization are highly specific in drug target binding, have good affinity to target molecules, and can accurately localize cellular target sites [42,43]. In the future, monoclonal IgY antibodies as therapeutic agents for diseases will focus on novel infectious diseases, rare diseases, and specific therapeutic applications.

## 5. IgY Applications in Human Health

### 5.1. The Mechanism of IgY Treatment and the Prevention of Human Diseases

IgY primarily functions through specific recognition and targeted blockade. It exerts its protective effects by neutralizing pathogens and toxins, regulating intestinal immunity, and inhibiting inflammation and allergies. Due to its safety and accessibility, IgY has significant potential for application in fields such as the treatment of gastrointestinal infections, enhancement of mucosal immunity, and prevention of allergies. The author elaborates on its mechanism of action from the following four perspectives:

Firstly, there is the specific neutralization of pathogens: IgY binds to antigens in a specific manner, thereby preventing viruses from binding to receptors on the surface of human cells and directly blocking the infection process [44,45]. For example, rotaviruses require binding to lactose receptors on intestinal epithelial cells to infect them. IgY can block this process and alleviate viral diarrhea [7]. Secondly, the inhibition of bacterial adhesion and colonization: IgY can bind specifically to adhesion proteins on the surface of bacteria, thereby blocking their binding to mucosal cells and preventing them from colonizing. This facilitates their expulsion from the body [46]. For instance, anti-*Helicobacter pylori* IgY reduces bacterial adhesion to the gastric mucosa, which aids in the treatment of gastritis. Thirdly, IgY regulates intestinal immunity and the mucosal barrier, maintaining microecological balance. It binds to pathogenic antigens in the intestine, reducing antigen stimulation of the intestinal mucosa and lowering mucosal inflammatory responses [47]. IgY can also promote the repair of intestinal epithelial cells, regulate the balance of intestinal microbiota, and enhance the mucosal barrier’s physical defensive capabilities. Fourthly, IgY has anti-inflammatory and anti-allergic properties that alleviate immune overactivation. It binds to self-antigens or inflammatory mediators, thereby preventing the formation of immune complexes and reducing neutrophil and macrophage aggregation and activation [48,49]. This alleviates tissue damage and relieves allergic symptoms.

### 5.2. Potential Human Health Benefits of IgY

The development of IgY for the prevention and treatment of human diseases is safe, non-invasive to animals, and highly efficient in antibody production. IgY will be able to overcome the limitations of traditional antibiotic treatments when microorganisms, such as bacteria, viruses, fungi, and parasites, change in ways that make targeted therapeutic drugs ineffective and lead to antibiotic resistance, which has become a global health emergency. Avian and mammalian species are more distantly related, and there are more differences between IgY and IgG, the most notable of which is that they do not bind to mammalian Fc receptors [50]. Therefore, it has stronger specificity and higher sensitivity in disease detection and prevention, and its application in human disease treatment and prevention research has gained considerable interest. It has been shown that prepared IgY-related antibodies exhibit significant potential in combating human diseases such as diabetes, HIV, tumors, inflammation, and viruses (Table 2).

## 6. Conclusions

In summary, this review systematically described the biological properties and action principles of IgY. It compared various methods of isolating and purifying antibodies, introduced methods of enhancing antibody activity, and focused on the current state of research on preventing and treating human diseases. However, IgY is a highly effective, high-quality, sustainable, non-antibiotic antibody that is widely available from a variety of sources. It is also much cheaper to obtain and more productive. Importantly, IgY has a low amino acid sequence homology with human immunoglobulin. It does not trigger human immune rejection or form immune complex precipitation. It can be used long term and is particularly suitable for infants, young children, and people with low immunity.

IgY shows significant potential in the fields of health maintenance and disease treatment, but its widespread use still faces many challenges. In the treatment of disease. IgY has demonstrated promising results in studies on antibacterial and antiviral infections. However, the pathways through which different pathogens infect the human body and the complex and diverse immune responses they trigger pose significant challenges. The precise and efficient exertion of its effects in the complex physiological environment of the human body requires further exploration. Furthermore, the pharmacokinetic characteristics, absorption, and metabolic processes of IgY in the human body remain understudied, hindering the precise determination of clinical dosages and treatment regimens. In terms of production, scaling up and standardizing IgY production is challenging. Factors such as chicken breed, immunization protocols, and separation and purification methods can significantly impact yield and quality. This results in inconsistent product quality that fails to meet stringent clinical and market requirements. Looking ahead, the continuous advancement of biotechnology means that further investigation into the mechanisms of IgY metabolism in the human body, combined with technologies such as gene editing and protein engineering, could enable structural modifications that enhance specificity and affinity. These modifications could improve therapeutic efficacy. On the other hand, the continuous optimization of production processes, precise control of chicken rearing and immunization procedures, development of new extraction and purification technologies, and creation of new biocompatible materials for incorporation could improve bioavailability and enhance activity, achieving the large-scale, standardized production of IgY. The use of IgY to prevent and treat human diseases has opened new avenues for the biopharmaceutical industry, offering economic and social benefits.

## Figures and Tables

**Table 1:** Methods of IgY isolation and purification.

Methods	Reagents or Devices	Steps or Principles	Purity	Recovery	Results	Reference
Inorganic precipitation	Ammonium sulfate	Hydrolysed with papain for 6 h; 45% saturated ammonium sulfate to remove low molecular weight peptides.	Fab 88.7% and Fc 90.1%.	No	Papain digest of IgY over 96%	[18]
Organic solvent precipitation	Octanoic acid	Ultrafiltration at pH 9.0 after addition of 2% caprylic acid in two batches.	97.9%	78.9%	99% activity	[19]
Polymer organic polymer precipitation	PEG precipitates	Isolation and purification of IgY at 6% PEG concentration;	81%	72.63%	Retention of activity 83.31%	[16]
		PBS was mixed with egg yolk and then 3.5% PEG, 8.5% PEG, 12% PEG were added.	80%	No	Average 60 mg IgY per egg	[20,21]
	PEG and ammonium sulfate union	3.5% PEG, 15% ammonium sulfate added to filtrate.	85%	No	Protein yield 6.8 mg/mL	[22]
	Poloxamer-PEG method	Adding skimming solution, then add PEG-6000 and mix with shaking.	92.71%	No	Total protein yield 30 mg/mL	[23]
Ultrafiltration	Ultrafiltration centrifuge	IgY forms dimers in 1.5 mol/L NaCl.	74–99%	80–85%	Ultrafiltration was more practical for industrial application	[24]
Chromatography	Ion exchange chromatography	PBS conditions with more net charge on the protein surface resulted in a more complete separation.	95%	94%	Retention of activity 73.77%	[16]
	Affinity chromatography	Epichlorohydrin and cyanuric chloride methods;highly stable affinity ligand named as ligand 8–6 purification by ligand.	92.1%	78.2%	Found 125 times increase in effective IgY	[25,26]
	Xanthan and carrageenan gums	Acidic natural gums remove lipoproteins.	98%	86%	Yield was 70 to 100 mg per egg	[27]
	Pectin	Dilution of egg yolks with 0.1% pectin in a 6-fold defatted solution.	83.3%	No	Protein yield 8.36 mg/mL	[28]
Water dilution	Proteins and lipids were separated by water dilution, and IgY was purified by sedimentation and ultrafiltration.		94%	No	Protein yield 9.8 mg/mL	[29]

**Table 2:** Potential benefits of IgY in preventing and treating human diseases.

Type of Diseases	Preparation of Specific IgY Antibodies	IgY Types	Potential Role	Reference
Diabetes mellitus	Human isomaltase (HISO) recombinant protein, emulsified with Freund’s adjuvant, was used to immunize laying hens for specific IgY. Antibodies were extracted via water dilution and sodium sulfate extraction.	Anti-HISO IgY	Targets HISO and inhibits alpha-glucosidase activity for the possible treatment of type II diabetes mellitus.	[51]
Human immunodeficiency virus (HIV)	Keyhole limpet hemocynin (KLH) by the glutaraldehyde method and chicken immunization with KLH-gp120 HIV fragment; antibodies were extracted by PEG precipitation.	Anti-HIV-gp120 IgY	Produced in chickens for diagnosis or treatment of patients with HIV.	[52]
Ovarian cancer	Chicken immunization with ovarian tumor-associated antigen 1 (OVTA 1) and generation of anti-OVTA 1 polyclonal IgY antibodies.	Anti-OVTA 1 polyclonal IgY	Ovarian cancer can be diagnosed using this method, which is also effective for the early screening of ovarian cancer.	[53]
Periodontitis	*Porphyromonas gingivalis* (Pg) and *Actinobacillus actinomycetemcomitans* (Aa) from dental plaque were used as antigens to immunize chickens for the preparation of anti-periodontitis-causing bacterial complex IgY, and antibodies were extracted by PEG precipitation.	Anti-periodontitis-causing bacterial complex IgY	Specific complex IgY inhibits the formation of Pg and Aa bacterial biofilms and exerts an antibacterial effect, which can be used for the targeted treatment of periodontitis.	[54,55]
Dental caries	*Streptococcus sobrinus* as the antigen, Freund’s complete adjuvant fully emulsified to immunize laying hens, IgY antibody purified by anion-exchange chromatography.	Anti *S. sobrinus* IgY	IgY inhibits the adhesion and acid production of *S. sobrinus*, reduces the abundance of oral *Streptococcus*, and effectively prevents dental caries.	[56,57]
Viral hepatitis type B(HBV)	Preparation of anti-HBV IgY for specific immunotherapy in laying hens immunized with hepatitis B vaccine (HepB).	Anti-HBV IgY	Showed strong antigen-specific binding activity as determined by ELISA.	[58]
Diarrhea	*Clostridium difficile* (CD) was detoxified with formaldehyde and mixed 1:1 with Freund’s complete adjuvant (FCA), and anti-CD IgY was prepared by immunizing laying hens, and the antibody was purified by ammonium sulfate precipitation.	Anti-CD IgY	Treating acute and recurring Clostridium difficile infection (CDI) in humans.	[59]
Hand-foot-and-mouth disease, (HFMD)	Anti-EV71 IgY was prepared by mixing EV71 strain as antigen with Freund’s incomplete adjuvant, 1:1 emulsion, immunizing laying hens and purifying the antibody by ammonium sulfate precipitation.	Anti-EV71 IgY	Recognizes the envelope proteins of EV71 and effectively inhibits viral infections.	[60]
COVID-19	Anti-SARS-CoV-2 IgY was prepared by immunizing laying hens with a mixture of formaldehyde-inactivated SARS-CoV-2 and Freund’s incomplete adjuvant emulsified in water, and antibodies were extracted by water dilution.	Anti-SARS-CoV-2 IgY	Strong activity in the upper respiratory tract and inhibition of SARS-CoV-2 infection with good safety and drug resistance.	[61,62]
Ebola virus (EBOV)	A thermostable therapeutic antibody against EBOV was developed modeled on the IgY; encoding the EBOV glycoprotein could enhance antibodies against EBOV.	Anti-EBOV IgY	Exhibits excellent thermostability and protective efficacy.	[63]
Human and simian rotaviruses	Inoculated Rhodia laying chickens with two or three doses of RVA combined with adjuvants or only adjuvants, prepared anti-RVA IgY.	Anti-RVA IgY	Latex beads performed concordant results with 75% ELISA and PCR-positive and 87.5% negative samples.	[64]

## Data Availability

Links to publicly archived datasets analyzed or generated during the study.
